# Protein structural context of cancer mutations reveals molecular mechanisms and candidate driver genes

**DOI:** 10.1016/j.celrep.2024.114905

**Published:** 2024-10-22

**Authors:** Diego Chillón-Pino, Mihaly Badonyi, Colin A. Semple, Joseph A. Marsh

**Affiliations:** 1 MRC Human Genetics Unit, Institute of Genetics and Cancer, University of Edinburgh, Edinburgh, UK

## Abstract

Advances in protein structure determination and modeling allow us to study the structural context of human genetic variants on an unprecedented scale. Here, we analyze millions of cancer-associated missense mutations based on their structural locations and predicted perturbative effects. By considering the collective properties of mutations at the level of individual proteins, we identify distinct patterns associated with tumor suppressors and oncogenes. Tumor suppressors are enriched in structurally damaging mutations, consistent with loss-of-function mechanisms, while oncogene mutations tend to be structurally mild, reflecting selection for gain-of-function driver mutations and against loss-of-function mutations. Although oncogenes are difficult to distinguish from genes with no role in cancer using only structural damage, we find that the three-dimensional clustering of mutations is highly predictive. These observations allow us to identify candidate driver genes and speculate about their molecular roles, which we expect will have general utility in the analysis of cancer sequencing data.

## Graphical abstract

**Graphical abstract:**
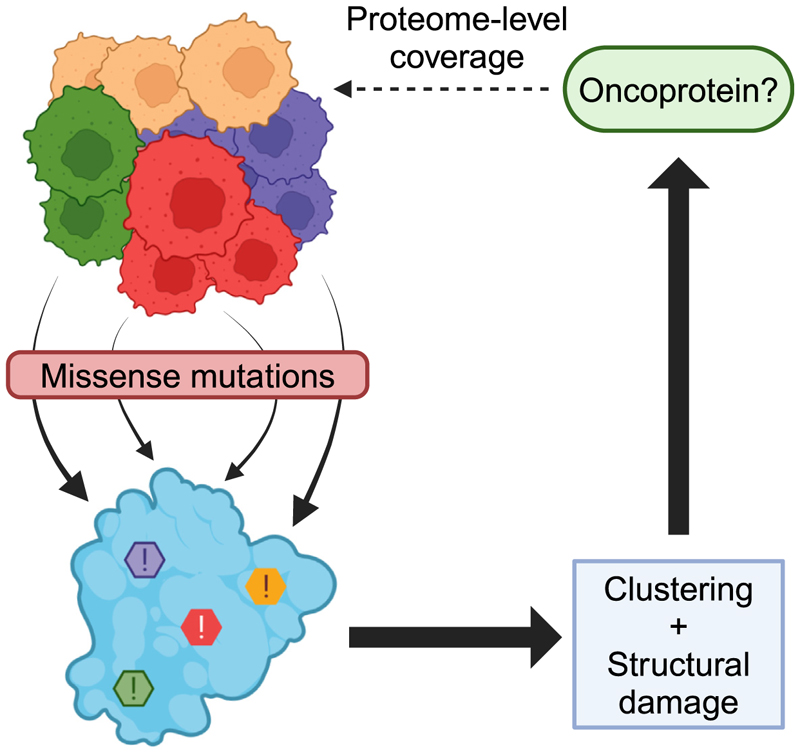

## Introduction

Tumor progression involves a complex process of genetic mutations accumulating over time, each potentially tipping the balance toward unchecked cell growth and the evasion of the body’s defense mechanisms. The proliferation of high-throughput sequencing platforms has revolutionized our ability to detect and catalog these genetic alterations, generating abundant mutation data in tumor profiling projects such as The Cancer Genome Atlas,^1^ the International Cancer Genome Consortium,^2^ and the Pan-Cancer Analysis of Whole Genomes^3^ and available in databases such as the Catalog Of Somatic Mutations In Cancer (COSMIC).^4^ Despite this wealth of data, deciphering the functional consequences of these mutations remains a formidable challenge.

Wide variations in the numbers and types of mutations are observed across different tumors. Most of these are thought to be passenger mutations that do not significantly impact tumor growth. However, a much smaller subset of mutations, known as driver mutations, play crucial roles in tumorigenesis and are selected for during tumor progression.^5,6^ In fact, most tumors are believed to possess just two to eight driver mutations.^7^ Identifying these driver mutations amid the chaotic landscape of genomic changes has been a pivotal challenge in cancer genomics and is crucial for understanding cancer mechanisms and for the development of targeted therapies.^8^ While some cancer drivers are characterized by significant structural alterations, such as chromosomal rearrangements, deletions, or duplications,^9^ many others result from small modifications within protein-coding regions. In particular, missense mutations, i.e., single nucleotide changes that results in single amino acid substitutions, have often been shown to play crucial roles in driving cancer.^10,11^ It can be difficult to distinguish drivers from passengers when considering missense mutations due to their oftensubtle protein-level effects, thus motivating considerable effort to develop computational predictive methods.^12–16^

Genes harboring cancer-driving mutations are often divided into two main classes: oncogenes and tumor suppressor genes (TSGs). Oncogenes tend to play crucial roles in promoting cell growth and cell division. In contrast, TSGs act as a safeguard by keeping cell growth and division in check, thereby protecting the organism from neoplasia.^17^ Numerous studies have shown that tumorigenesis is largely driven by mutations resulting in gain of function of oncogenes along with the loss of function of TSGs.^17^ The intrinsic differences in the molecular mechanisms of mutations in these two categories of genes are evident in distinct mutational patterns,^18^ hotspots,^19–21^ and patterns of selection.^22^

Recently, we investigated the protein structural differences between pathogenic missense mutations, primarily associated with Mendelian disorders, that act via gain- vs. loss-of-function molecular mechanisms.^23^ In particular, we observed that gain-of-function mutations tend to have much milder effects on protein stability and interactions within protein complexes than recessive or haploinsufficient missense mutations associated with loss of function. In addition, gain-of-function mutations showed a much greater tendency to cluster within three-dimensional protein structures. This suggested that the molecular mechanism underlying pathogenic mutations in a gene could potentially be predicted by considering protein structural context.

The classification of cancer genes into TSGs and oncogenes closely mirrors the terminology used in rare genetic disease, where most pathogenic mutations can be classified as being associated with loss-of-function or gain-of-function mechanisms.^24,25^ Given that mutations in TSGs and oncogenes are often assumed to act via loss and gain of function, respectively, we reasoned that a similar large-scale analysis of cancer-associated mutations could provide insight into their cancer-driving mechanisms. Previous work has shown TSG mutations to be more structurally damaging than oncogene mutations across relatively small sets of cancer-associated mutations.^15,20,26^ Furthermore, a number of studies have investigated patterns of mutational clustering and hotspots in oncogenes and TSGs.^3,19,21,26–32^

In this study, we have investigated the protein structural context of cancer-associated missense mutations, taking advantage of the many protein and protein complex structures that have now been experimentally determined^33^ and the availability of computationally predicted structural models across the entire human proteome.^34^ While, collectively, cancer-associated mutations show only a small tendency to be structurally damaging, we observed striking structural differences between driver mutations in TSGs compared to oncogenes. Moreover, we are able to identify many known TSGs as those that are most strongly enriched in structurally damaging mutations. In contrast, while oncogene mutations tended to be structurally mild, they showed strong clustering within three-dimensional protein structures. Finally, we use both structural perturbation and clustering to identify genes that exhibit the characteristic properties of TSGs and oncogenes. Overall, we show that consideration of protein structure can provide new insights into the molecular mechanisms underlying cancer-associated mutations and can potentially identify candidate cancer-driving genes.

## Results

### Cancer-associated missense mutations are enriched for structurally damaging mutations

To investigate the protein structural context of cancer-associated mutations, we used missense mutations from the Cancer Mutation Census (CMC),^35^ an ongoing project branching from the COSMIC project,^4^ which we refer to as the “*cancer-all*” set. These are somatic variants that have been identified in tumor samples, but they are not necessarily important for tumorigenesis; we expect many of these to be passenger mutations. In addition, we selected a subset of these mutations observed to occur multiple times in the CMC, referred to as “*cancer-recurrent*,” and a subset annotated for their relevance in cancer within the Cancer Gene Census (CGC), referred to as the “*cancer-driver*” set.^35^ For comparison, we included missense mutations classified as pathogenic and likely pathogenic in ClinVar^36^ across all human protein-coding genes as the “*pathogenic*” set and non-pathogenic missense variants observed in the human population from gnomAD v2.1^37^ as the “*putatively benign*” set, as done previously.^23,38^ Next, we mapped missense mutations from the four groups to experimentally determined protein structures from the Protein DataBank (PDB)^33^ and to AlphaFold2 computationally predicted models^34^ (Table 1).

**Figure 1:**
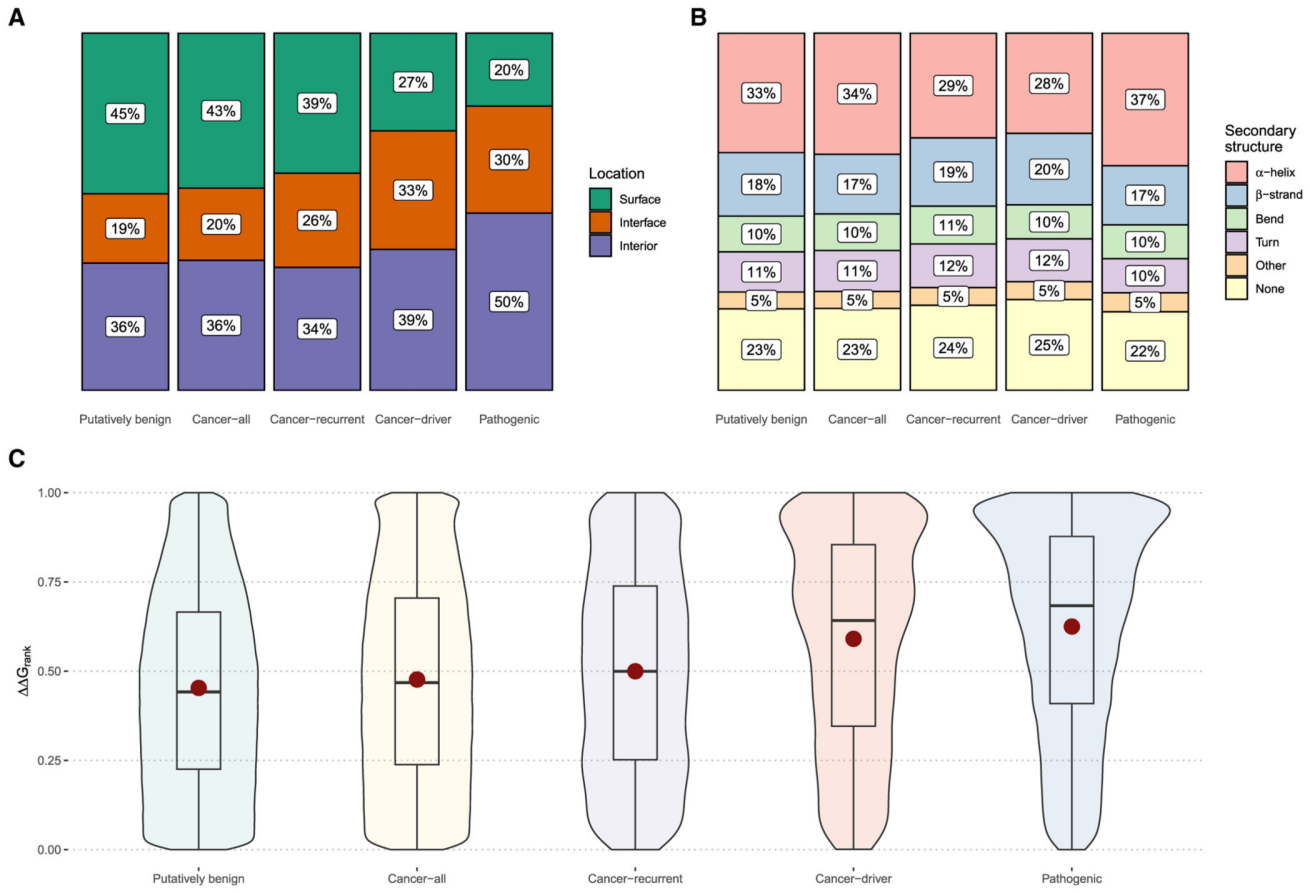
Protein structural properties of different classes of missense mutations Putatively benign mutations are those observed in the human population (gnomAD) without a reported disease association. Cancer-all represents all mutations from the CMC. Cancer-recurrent comprises only recurrent mutations from the CMC (recurrence ≥7). Cancer-driver mutations are the subset of mutations annotated for their direct role in cancer. Pathogenic mutations are those annotated as pathogenic or likely pathogenic in ClinVar. (A) Locations of mutations within protein structures present in the Protein DataBank (PDB), split into surface, interface, and interior positions, as defined previously.^83^ (B) Occurrence of mutations within different types of secondary structures. (C) Violin plot distributions of predicted structurally damaging effects, as measured by the ΔΔG_rank_ metric, whereby 0 represents the mildest possible single amino acid substitution in a protein, 1 represents the most damaging, and random mutations would be expected to have a mean of 0.5. The mean value of each distribution is represented with a red dot for the datasets. All comparisons between group pairs proved to be highly significantly different, with *p* values <1.5 × 10^−8^ according to Wilcoxon tests. Boxes represent the interquartile range (IQR), with the line inside indicating the median, and the whiskers extend to the smallest and largest values within 1.5 times the IQR. Equivalent analyses based on AlphaFold2 models are shown in Figure S1.

In Figure 1A, we investigate the locations of mutations from the different groups within PDB structures, classifying each mutation based on whether it occurs in the protein interior, on the surface, or at an interface. It is well known that pathogenic mutations tend to be enriched at protein interior and interface residues, as mutations at these positions are more likely to be disruptive to protein structure.^39,40^ This is confirmed here, with 80% of the *pathogenic* set of mutations occurring at interior and interface positions, compared to only 55% of the *putatively benign* mutations. Interestingly, the *cancer-all* mutations are very similar to the *putatively benign* mutations in distribution, with only a small, albeit highly significant, enrichment at interior and interface positions (57%, odds ratio [OR] = 1.07, *p* = 1.63 × 10^−97^, Fisher’s exact test). In contrast, the *cancer-recurrent* and *cancer-driver* groups are intermediate, with 61% and 73% occurring at interior and interface residues, respectively. Notably, the *cancer-driver* group is slightly enriched in interface mutations (33%) compared to the *pathogenic* group (30%, OR = 1.17, *p* = 5.58 × 10^−4^), consistent with previous work demonstrating enrichment of cancer-associated mutations at specific protein interfaces.^41–43^ Similar patterns are observed when using AlphaFold models (Figure S1), although for these, we can only classify interior and surface positions.

We also considered the distribution of mutations across secondary structure types within PDB protein structures (Figure 1B), given the observation that α helices and β strands have different mutational sensitivities.^44^ Interestingly, while the *pathogenic* mutations are significantly enriched at α helices (37%) compared to the *putatively benign* (33%, OR = 1.18, *p* = 2.96 × 10^−40^, Fisher’s exact test) and *cancer-all* groups (34%, OR = 1.15, *p* = 2.48 × 10^−30^), the *cancer-driver* set is relatively deficient, with 28% of mutations occurring at α-helical positions (OR = 1.32, *p* = 3.34 × 10^−10^ vs. *cancer-all*). In addition, while all other mutation groups are nearly identical across other secondary structure classes, the *cancer-driver* group is enriched at β strand positions (20% vs. 17% for *cancer-all*, OR = 1.25, *p* = 1.5 × 10^−5^) and at regions without regular structure (25% vs. 23% for *cancer-all*, OR = 1.14, *p* = 3.53 × 10^−3^). Overall, it appears that, while the differences in secondary structure between the mutation groups are modest, the *cancer-driver* mutations have some distinct properties relative to both the *pathogenic* and *cancer-all* groups.

Finally, we modeled the effects on protein stability by using FoldX^45^ to calculate ΔΔG values. Previous work has demonstrated that FoldX outperforms other stability predictors in the identification of disease mutations^46^ and shows higher correlations with deep mutational scanning data.^47^ For easier visualization and comparison of ΔΔG values, here, we introduce a rank normalized metric we call ΔΔG_rank_. First, for a given human protein, we use FoldX to calculate ΔΔG values for all possible missense mutations (i.e., all single amino acid substitutions possible via single nucleotide changes). These are then sorted based on absolute ΔΔG values, as absolute values have been found to show slightly stronger correspondence with disease than raw ΔΔG.^46^ These are then normalized from 0 to 1, with 0 representing the mildest possible missense mutation for a protein in terms of its effect on protein stability, and 1 representing the most structurally damaging. For the PDB structures, we compute the ΔΔG_rank_ using full complex structures, when available, as the inclusion of intermolecular interactions considerably improves the explanatory value of ΔΔG.^23,47^

We have introduced ΔΔG_rank_ for two main reasons. First, FoldX tends to output ΔΔG values with a skewed distribution that can make their visualization and interpretation difficult. The large majority of FoldX ΔΔG values are lower than ∼3 kcal/mol, and the optimal threshold for identifying pathogenic mutations is ∼1.5 kcal/mol.^46^ However, FoldX will occasionally output extreme outliers. Thus, when plotted, the most informative range of the ΔΔG distribution will often only take up a small fraction of the scale. Second, different proteins can have very different intrinsic propensities for destabilizing mutations, which can make comparisons of ΔΔG values between different proteins difficult. The ΔΔG_rank_ rank scale benefits from being highly interpretable, with a mean value of exactly 0.5 across all possible mutations in a protein. Thus, in the absence of any selection, an average ΔΔG_rank_ value of ∼0.5 would be expected for a set of random mutations.

In Figure 1C, we compare the distributions of ΔΔG_rank_ values calculated from PDB structures for different mutation datasets. Consistent with previous observations, *pathogenic* mutations are more structurally disruptive (mean ΔΔG_rank_ of 0.62) than *putatively benign* mutations (mean ΔΔG_rank_ of 0.45). Interestingly, both the *cancer-all* and *cancer-recurrent* mutations are very similar to what would be expected for random missense changes, with mean ΔΔG_rank_ of 0.48 and 0.50, respectively. In contrast, mutations from the *cancer-driver* set have a mean ΔΔG_rank_ of 0.59, suggesting that they are enriched in structurally damaging mutations but are overall significantly milder than the pathogenic set. A very similar pattern is observed using the AlphaFold models (Figure S1).

It is important to emphasize that our analysis is based on computationally predicted effects on protein stability, and these can potentially be quite different from experimental measurements. Recent high-throughput experimental approaches have been able to measure effects on protein stability on a much larger scale than has previously been feasible.^48,49^ While these studies still do not provide nearly enough coverage of human proteins for the analyses performed here, in Figure S2, we compare computationally predicted ΔΔG used in this study to all of those that had experimental values available in a recent high-throughput study based on cDNA display proteolysis.^48^ This is consistent with previous work showing that, while ΔΔG predictors like FoldX are good at capturing overall trends for large sets of mutations, experimental and computational ΔΔG can vary widely for individual mutations.^50^

### Tumor suppressor proteins show distinct patterns of structural damage compared to oncogenes

Our initial results show that, overall, cancer-associated missense mutations are only slightly more damaging at a protein structural level than putatively benign variants observed in the human population, consistent with the idea that the mutational landscape of tumors is dominated by passengers. Interestingly, however, even those mutations with evidence for being cancer drivers are still milder than pathogenic ClinVar mutations, suggesting that, in general, cancer-driving missense mutations tend to have weaker effects on protein structure than mutations that cause Mendelian disorders. Our previous work found that mutations that cause disease via gain-of-function mechanisms tend to induce much smaller perturbations in protein stability than those that act via a loss of function.^23^ Therefore, we hypothesized that the milder protein structural effects of cancer-driving mutations are due to a greater tendency to be associated with gain-of-function effects.

**Figure 2:**
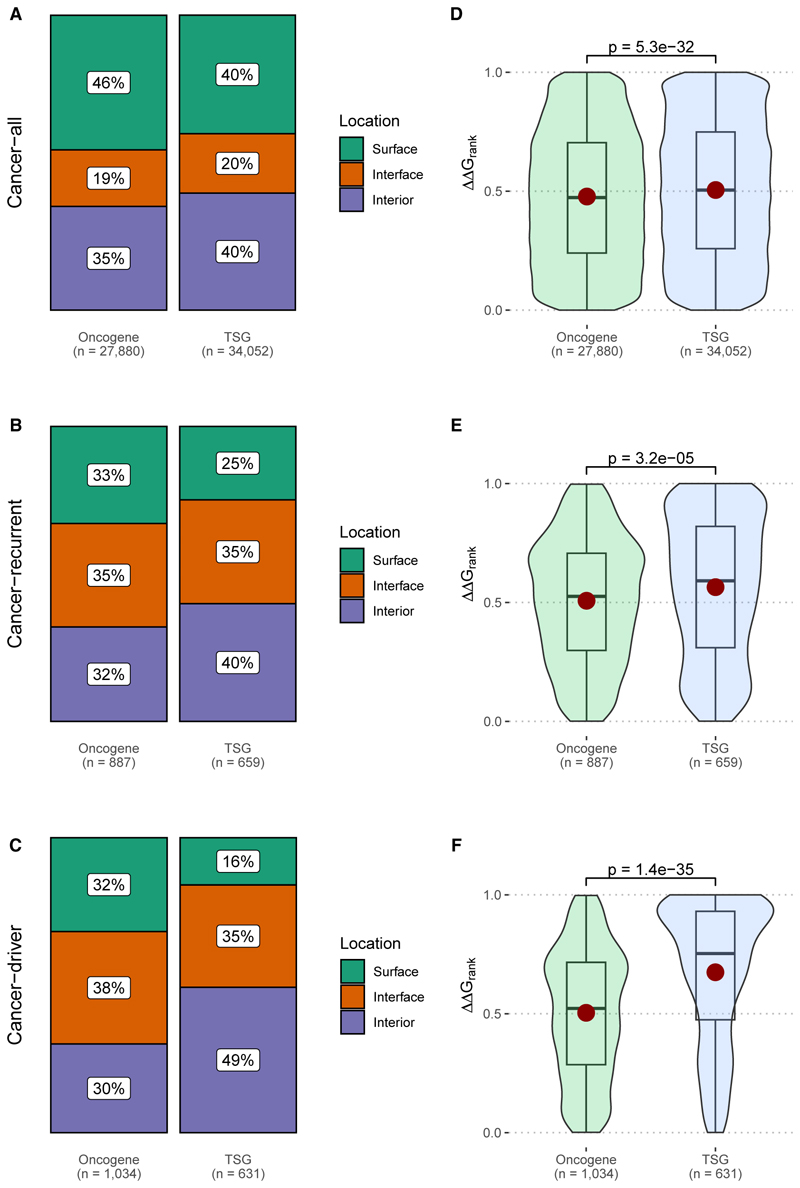
Protein structural properties of cancer mutations in oncogenes and tumor suppressors (A and B) Locations of all cancer mutations (A) within PDB structures (cancer-all dataset) and (B) distributions of predicted structural damage, as represented by ΔΔG_rank_ values. (C and D) Locations of recurrent cancer mutations (recurrence ≥7) (C) within PDB structures (cancer-recurrent dataset) and (D) distributions of predicted structural damage, as represented by ΔΔG_rank_ values. (E and F) Locations of cancer mutations (E) with an annotated role in cancer (cancer-driver dataset) and (F) distributions of ΔΔG_rank_ values. The *p* values are calculated using Wilcoxon tests. Boxes represent the IQR, with the line inside indicating the median, and the whiskers extend to the smallest and largest values within 1.5 times the IQR. Equivalent analyses based on AlphaFold models are shown in Figure S3.

To address this, we used classifications of known oncogenes and TSGs from the CGC. First, we compare the location distributions in oncogenes vs. TSGs for the different mutation groups (Figures 2A–2C). All three show significant enrichments in mutations at interior positions for the TSGs relative to the oncogenes, consistent with their expected tendency to be more structurally damaging. This trend is strongest for the *cancer-driver* group and weakest for *cancer-all*. The intermediate nature of the *cancer-recurrent* group suggests that it contains a mixture of both driver and passenger mutations. Interestingly, the fraction of mutations occurring at interface positions is very similar for oncogenes vs. TSGs across the three groups. This suggests that occurrence at interior positions is a hallmark of cancer-driving mutations in TSGs, consistent with their expected tendency to be structurally damaging. In contrast, while occurrence at interface positions appears to be strongly associated with cancer-driving activity, this propensity is similar for TSG and oncogenes, presumably because interface mutations can have either loss- or gain-of-function effects.^23^ Similar patterns are observed using AlphaFold models in Figure S3.

Next, we considered the structural impact of missense mutations as measured by ΔΔG_rank_ values (Figures 2D–2F). For *cancer-all*, the overall difference is small but significant, with a mean of 0.50 for the TSGs and 0.48 for the oncogenes. For *cancer-recurrent*, the trend is slightly stronger, with a mean of 0.56 vs. 0.51 for TSGs vs. oncogenes. However, for the *cancer-driver* group, the difference is striking, with a mean of 0.67 for TSGs vs. 0.50 for oncogenes. Similar trends are observed for AlphaFold models (Figure S3). Thus, the cancer-driving missense mutations in TSGs tend to be even more disruptive than the pathogenic mutations. This supports the idea that the structurally milder nature of the cancer-driving mutations, when considered collectively, is a consequence of their lower tendency to be associated with loss-of-function molecular mechanisms, due to the oncogenic nature of many cancer-driving mutations. In other words, the balance between loss-of-function vs. gain-of-function effects appears to be shifted toward gain of function for cancer-driving mutations compared to pathogenic mutations associated with genetic disease.

## Gene-level enrichments in damaging mutations reveal known tumor suppressors

**Figure 3:**
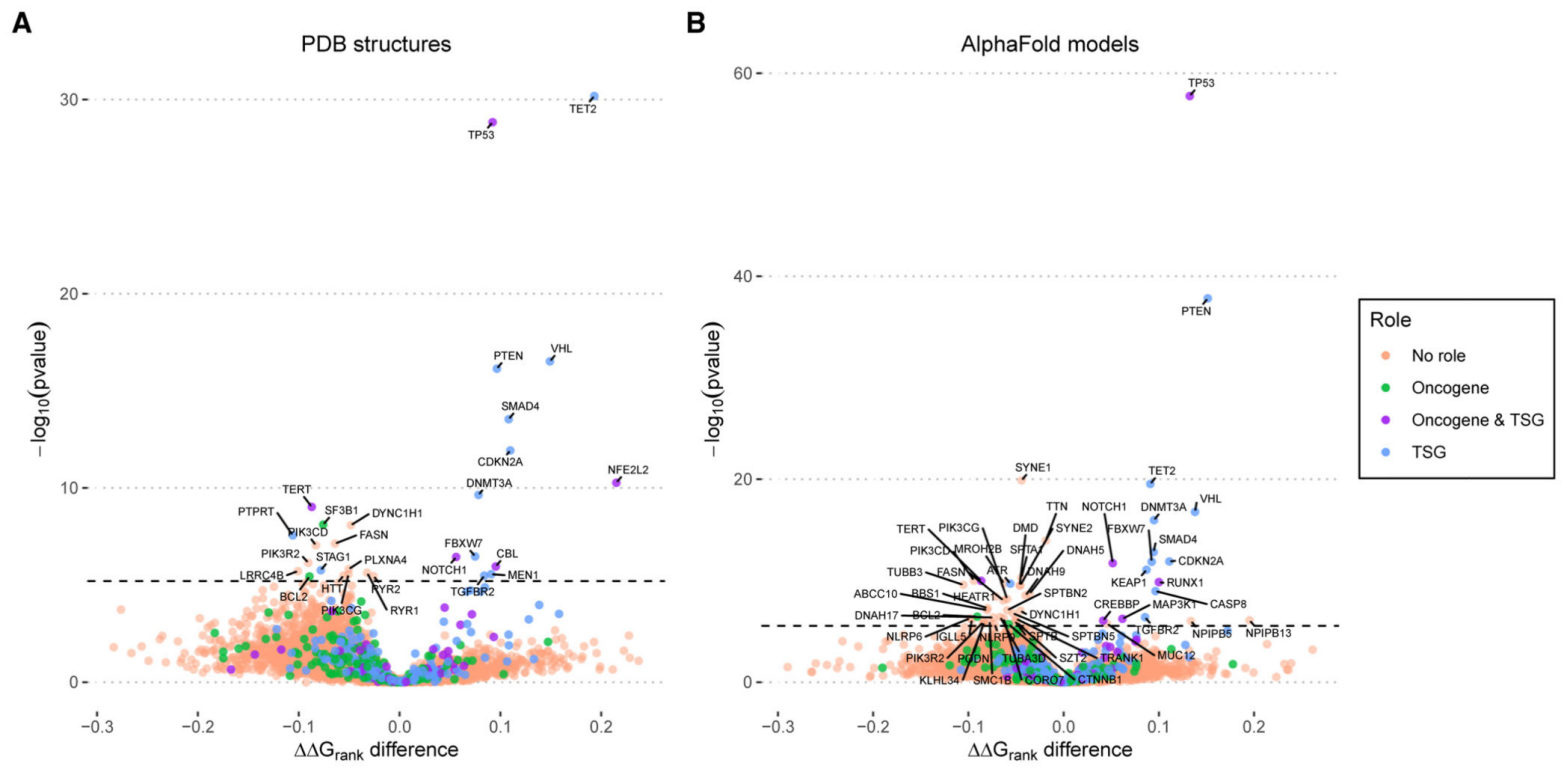
Gene-level enrichment in structurally damaging and structurally mild cancer-associated missense mutations For each of the 8,402 human protein-coding genes with a PDB structure (A) or 17,905 with an AlphaFold model (B), we plot the difference between the mean ΔΔG_rank_ for mutations observed in the cancer-all dataset and for other possible missense mutations not present in the dataset. Proteins with positive ΔΔG_rank_ difference values are enriched in structurally damaging mutations, in that the average of the observed cancer-associated mutations is more destabilizing than the average of the possible but unobserved mutations. In contrast, proteins with negative ΔΔG_rank_ difference values are enriched in structurally mild mutations, in that the average of the observed mutations is less destabilizing. The *p* value of this difference is calculated with the Wilcoxon test. The horizontal dashed bar represents the threshold for a statistically significant values, as result of a Bonferroni correction: 6.26 × 10^−6^ for the PDB structures and 2.88 × 10^−6^ for the AlphaFold models. Proteins are colored based on their classified role in the Cancer Gene Census (CGC).

Although the differences in structural damage between oncogenes and TSGs were minimal in the *cancer-all* dataset when considering these groups collectively, we wondered if we could identify specific protein-coding genes enriched in structurally damaging or structurally mild mutations. For each protein, we calculated the difference between the mean ΔΔG_rank_ for the *cancer-all* mutations and the mean ΔΔG_rank_ for all other possible (but not observed) missense mutations. Proteins with a ΔΔG_rank_ difference greater than 0 are relatively enriched in mutations that are structurally damaging compared to what would be expected if mutations occurred randomly without selection, whereas those with a negative ΔΔG_rank_ are enriched in structurally mild mutations. We show ΔΔG_rank_ difference values across all proteins based on PDB structures (Figure 3A) and AlphaFold models (Figure 3B) as a volcano plot, where the Wilcoxon *p* value represents the significance of the difference between observed and unobserved mutations.

Proteins on the right sides of the volcano plots are enriched in structurally damaging mutations. For those with statistically significant *p* values (above the dashed line), this implies that there has been selection for damaging mutations. In other words, structurally damaging mutations in these proteins are expected to drive cancer. Remarkably, all of the proteins with the most significant enrichments in structurally damaging mutations, using both PDB structures and AlphaFold models, have known tumor suppressor activity, including TET2, TP53, VHL, PTEN, SMAD4, CDKN2A, NFE2L2, and DNMT3A. Even below the strict statistical significance threshold (*p* < 6.26 × 10^−6^), which accounts for multiple testing, there is a clear enrichment of known TSGs on the right side of the plot, suggesting that this approach could be useful for identifying genes with putative tumor suppressor activities.

Interestingly, while the most significantly enriched proteins occur on the right side of the plots, reflecting strong selection for structurally damaging mutations in certain proteins, there are far more proteins with negative ΔΔG_rank_ difference values. To some extent, this is likely to reflect positive selection for cancer-driving mutations that are not structurally damaging, e.g., gain-of-function mutations. Indeed, the two most significantly enriched proteins in the PDB analysis, TERT and SF3B1, have known oncogenic activity. However, purifying selection against structural damage mutations may be an even greater contributor to the enrichment in structurally mild mutations. In oncogenes, damaging mutations that cause a loss of function are likely to be strongly selected against. In addition, proteins that have no specific role in cancer, but which are important for cellular growth or viability, are also likely to experience selection against structurally damaging loss-of-function mutations. Thus, a statistical enrichment in structurally mild mutations does not necessarily imply an oncogenic role.

Several proteins classified as TSGs also appear on the left sides of the plots, including PTPRT, STAG1, and ATR. This suggests that the cancer-driving effects of mutations in these proteins are unrelated to loss of function induced by destabilization. One possible explanation is that damaging mutations in these TSGs disrupt other aspects of function, such as protein interactions. The AlphaFold analysis, being based only on monomeric models, will not account for interaction-disrupting effects. While the PDB analysis does include many experimentally determined protein complex structures, these do not include all biologically relevant interactions. Thus, some mutations that have little apparent structural impact in our analysis may be damaging to specific protein interactions.

Finally, we wondered whether ΔΔG_rank_ difference might show some correspondence with sequence conservation, given that previous work has observed distinct evolutionary patterns in cancer-associated genes.^51^ Overall, we observe only a very weak negative correlation between ΔΔG_rank_ difference values and gene-level evolutionary conservation, such that proteins enriched in structural damage show a slight tendency to be less conserved (Figure S4). Furthermore, ΔΔG_rank_ difference values show much more highly significant differences between TSGs and other groups than conservation.

### Oncogene mutations show characteristic clustering in three-dimensional space

**Figure 4:**
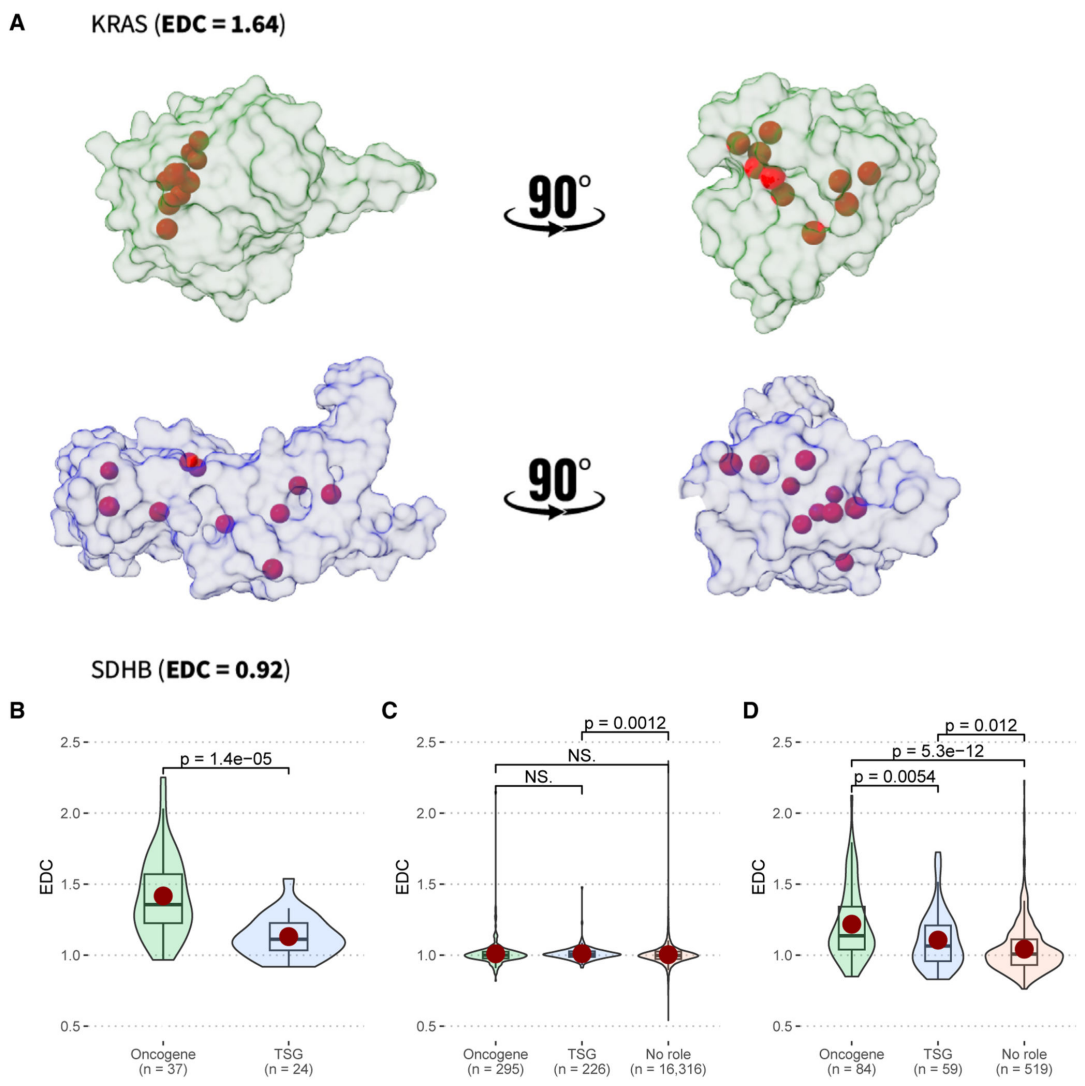
Clustering of cancer-associated mutations in three-dimensional space (A) Location of cancer-driver mutations for an oncogene (KRAS) and TSG (SDHB), highlighting their remarkably different clustering, as reflected by the high Extent of Disease Clustering (EDC) value for KRAS and low EDC value for SDHB. (B) Distribution of EDC values calculated from mutations from the cancer-driver dataset, split into those cancer-associated genes classified as having oncogene activity and those genes only classified with TSG activity. (C) Distribution of EDC values calculated from the cancer-all dataset. (D) Distribution of recurrent EDC values calculated from the cancer-driver dataset. The *p* values are calculated using Wilcoxon tests. Boxes represent the IQR, with the line inside indicating the median, and the whiskers extend to the smallest and largest values within 1.5 times the IQR.

Previously, we introduced a novel protein structural metric, the Extent of Disease Clustering (EDC), that showed remarkably strong discrimination between genes associated with gain-of-function vs. loss-of-function mechanisms.^23,52,53^ EDC is a simple measure that quantifies the clustering of diseases mutations within a three-dimensional protein structure. An EDC value greater than one indicates that disease mutations tend to be close to each other, while a value of one would be expected if the disease mutations were randomly distributed throughout the protein. Therefore, given the association of oncogenes and TSGs with gain-of-function and loss-of-function mutations, respectively, we wondered whether EDC values would also be useful for the identification of cancer-associated genes and for the discrimination between oncogenes and TSGs. To illustrate, in Figure 4A, we show two examples of mutation distributions. For the oncogene KRAS, known cancer-driving missense mutations are observed to be highly clustered on the protein structure, resulting in a high EDC value of 1.69. In contrast, for the tumor suppressor SDHB, the known driver mutations are spread throughout the protein, resulting in a low EDC of 0.85.

First, we considered EDC values for known cancer-driving mutations from the *cancer-driver* dataset (Figure 4B), revealing a strong, highly significant tendency for the mutations in oncogenes to be more clustered than those from TSGs. We considered proteins with mutations present at five or more residues, the same threshold as we have used in recent studies,^52,53^ although our results are similar across different minimum residue thresholds (Figure S5A). The oncogenes had a median EDC of 1.48, compared to 1.25 previously observed for gain-of-function mutations.^23^ The TSGs had a mean EDC of 1.13, similar to the value of 1.09 observed for loss-of-function missense mutations in autosomal dominant genes.

We next calculated EDC values from the *cancer-all* dataset, thus considering the clustering properties of driver and passenger mutations collectively. We observed similar distributions of EDC values for oncogenes and TSGs, as well as for genes with no known role in cancer (Figure 4C). In fact, the large majority of EDC values are very close to one. Thus, it appears that the large number of passenger missense mutations in this dataset likely obscures our ability to detect any signs of clustering using the EDC metric, even in known oncogenes and TSGs.

The clustering of known driver mutations is of little utility for identifying candidate cancer-driving genes. Therefore, we next limited our analysis to the *cancer-recurrent* dataset, allowing us to include far more genes than with the *cancer-driver* dataset, many of which have no known role in cancer (Figure 4D). We observe significantly higher recurrent EDC values in the oncogenes compared to TSGs, although the extent of clustering is somewhat less pronounced than observed for the driver mutations alone, with a mean EDC of 1.16 for the oncogenes compared to 1.07 for the TSGs. We also observe EDC values in both oncogenes and tumor suppressors to be significantly higher compared to genes with no known cancer role, suggesting that some degree of clustering does occur in TSGs, but to a less extent than in oncogenes. This may be related to damaging missense mutations being more likely in certain regions of tumor suppressors, e.g., around functionally important sites, and it is consistent with the previous observation of clustering in other TSGs.^54,55^ We also note that our results are robust to different recurrence thresholds (Figure S5B).

### Identification of candidate tumor suppressors and oncogenes using protein structural information

**Figure 5:**
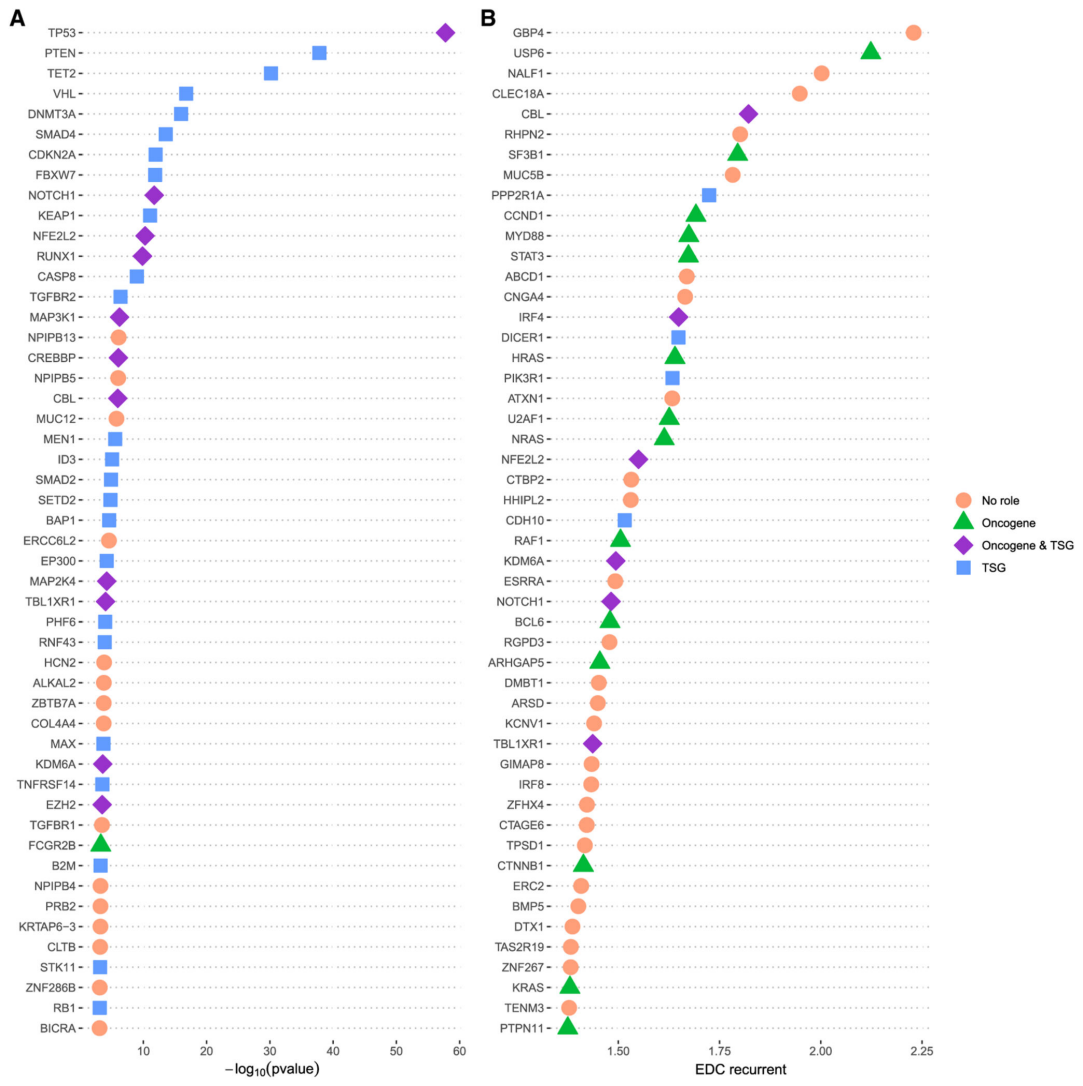
Prioritization of putative cancer-driving genes (A) Top-50 human protein-coding genes enriched in structural damaging mutations, i.e., those with positive ΔΔG_rank_ difference values. For these proteins, the observed missense mutations in the *cancer-all* dataset are significantly more destabilizing than the possible but unobserved mutations. The *p* values from both the PDB and AlphaFold analyses in Figure 3 are included, with the most significant value from either analysis selected for each protein to be used in this ranking. (B) Top-50 human protein-coding genes with the highest recurrent EDC values, based on recurrent mutations from the *cancer-all* dataset, as in Figure 4D.

As both structural disruption and clustering of missense mutations appear to be predictive of genes that have roles in cancer, we explored the potential of these properties to prioritize candidate TSGs and oncogenes. First, in Figure 5A, we show the 50 proteins most significantly enriched in structurally damaging mutations, combining both the PDB and AlphaFold analyses. The 15 highest ranked proteins, and 34 out of the top 50, have known tumor suppressor activities, demonstrating the strong potential of this approach for identifying putative cancer-driving genes.

Next, we explored those proteins with no role in cancer classified in the CGC. The most significantly enriched of these proteins, NPIPB13 and NPIPB5 (ranking 16^th^ and 18^th^ overall, respectively), are both members of the primate-specific nuclear pore complex interacting protein (NPIP) family. Very little is known about the normal biological function of this family or its potential role in human disease. One recent study has linked NPIPB5 expression to prognosis and patient survivability in renal cell carcinoma,^56^ while another found NPIPB13 expression to be weakly associated with microvascular invasion in hepatocellular carcinoma.^57^ The closely related NPIPB4 is also listed in Figure 5A, ranking 43^rd^ overall. While the previous limited known biological role or cancer association for these proteins may argue against a tumor suppressor function, we find it interesting that similar patterns of enrichment in damaging mutations are observed across all three of these closely related homologs, suggesting that it could be worthy of further investigation.

MUC12 was the next most significantly disrupted gene with no cancer classification, ranking 20^th^ overall. Previously, its expression was found to be significantly lower in colorectal cancer tissues, indicative of potential tumor suppressor activity.^58^ In contrast, other research suggested that MUC12 was overexpressed in renal cell carcinoma.^59^ Its high significance is influenced by its long length (5,478 amino acids), and the effect size is relatively small, but our results suggest that there may be a tendency of structurally damaging missense mutations in this protein to drive cancer.

ERCC6L2 ranks 26^th^ overall, and it appears to be involved in DNA repair processes,^60^ with recessive protein null mutations being associated with bone-marrow-failure syndrome.^61^ It also possesses two domains that commonly occur in known TSGs: the *Helicase_C* domain is found in nine well-established TSGs, and the *SNF2-rel_dom* domain is found in three. Notably, patients with bone-marrow-failure syndrome have also been observed to be at a high risk of developing acute myeloid leukemia.^62^ Thus, ERCC6L2 seems to be a strong candidate as a putative tumor suppressor.

ZBTB7A, ranking 34^th^ overall, is a transcriptional repressor involved in cell proliferation and differentiation.^63^ The protein has an N-terminal BTB dimerization domain. BTB domains are known to be strong drivers of cotranslational assembly,^64^ a process that lessens the likelihood of observing a disease mechanism other than loss of function.^52^ Consistently, heterozygous variants in ZBTB7A have been linked to a neurodevelopmental phenotype and are suggested to cause loss of function.^65^ One such variant, D452N, has been identified in an individual with severe hematological issues^65^ and as a recurrent somatic mutation (*n* = 4) in malignant hematopoietic and lymphoid tissues.^66^ Another recurrent (*n* = 8) somatic mutation, K424T, observed in multiple adenocarcinoma samples, lies close to D452 within two adjacent zinc-finger domains that map to the DNA interface in the crystal structure.^67^ A recent study concluded that K424T results in a significant reduction in transcriptional activity, further supporting a loss-of-function mechanism.^68^ While the gene has not yet been classified with a cancer role in the CGC, it is increasingly being recognized as a potential cancer driver.^69^ For example, somatic loss-of-function mutations in ZBTB7A cause elevated glycolysis in human cancer,^70^ and loss of one copy or the C-terminal zinc-finger domains has been associated with acute myeloid leukemia.^71^ Thus, while in the case of ZBTB7A our method does not offer a completely novel target, it nevertheless further supports its role in cancer and provides validation of our approach.

We also investigated the use of recurrent EDC values to identify cancer-driving genes. In Figure 5B, we show the top-50 proteins with the highest recurrent EDC values, as used in Figure 4C. Of these, 24 are classified as known cancer-driving genes in the CDC, with 14 being oncogenes, 6 having both oncogene and TSG activity and 4 being TSGs. This is consistent with our observation that, while oncogenes show the highest degree of mutation clustering, there is also significant clustering in TSGs. Although known cancer drivers are not quite as highly enriched as for the structural damage analysis, this does appear to be a promising strategy for identifying putative cancer-driving genes.

GBP4 shows the strongest clustering among all proteins, with a recurrent EDC value of 2.23. Examination of the underlying mutational data shows a cluster of highly recurrent mutations at a small stretch from residues 541 to 551 in the coiled-coil domain, supportive of a potential cancer-driving activity associated with this region. While the precise role of GBP4 in cancer is still unclear, there has been some previous work suggesting its involvement^72^; in particular, it has been observed to be upregulated in certain tumor types.^73^

Another of our top hits with no cancer classification, CNGA4, has a recurrent EDC of 1.66 and features two domains, transmembrane ion transport domain (*Ion_trans*) and cyclic nucleotide-monophosphate binding domain (*cNMP_binding*), which are also found in two known oncogenes, CACNA1D and PRKAR1A, respectively. Recurrent *cancer-all* mutations in CNGA4 are limited to 5 distinct residues that exhibit high clustering in the *Ion_trans* domain. Leveraging the previously modeled tetrameric structure of CNGA4,^74^ our analysis revealed that the mutations cluster at the channel pore, suggesting a potential gain-of-function effect. Since CNGA4 is important for transduction of odorant signals,^75^ it is possible that mutant proteins are advantageous to chemotaxis-mediated processes in cancer.^76^

Four proteins occur in the top 50 for both enrichment in structurally damaging mutations and recurrent EDC: NOTCH1, KDM6A, CBL, and TBLXR1. Interestingly, all of these are classified as both oncogenes and TSGs in the CGC. Thus, the combination of high structural damage and clustering in three-dimensional space represents a strong indicator of genes with both oncogenic and tumor suppressor activity.

As discussed earlier, enrichment in structurally mild mutations is not nearly as predictive of cancer association as enrichment in damaging mutations. In Figure S6, we show the top-50 genes most significantly enriched in structurally mild mutations (i.e., those from the left side of the volcano plots in Figure 3). Of these, only eight have a known cancer role, including four oncogenes, three TSGs, and one with both activities. Many of the proteins most significantly enriched for mild mutations are of large size, including very long proteins like SYNE1 and TTN with modest negative ΔΔG_rank_ values but highly significant *p* values in the AlphaFold analysis due to their large numbers of mutations. Similarly, the very large calcium channels RYR1 and RYR2 are both significantly enriched in the PDB analysis, but it seems unlikely they represent true cancer drivers. Interestingly, the beta-tubulin gene TUBB3 is also highly enriched in structurally mild mutations. Given that TUBB3 expression is strongly associated with resistance to anti-microtubule chemotherapeutics,^77^ this could possibly result from selection against damaging mutation in patients who have undergone chemotherapy or selection for mutations that confer greater resistance.

## Discussion

This study investigates the protein structural context of cancer-associated missense mutations. A major challenge associated with this is the fact that datasets of cancer mutations are inevitably dominated by passenger mutations. Thus, although we find that properties of known cancer-driving mutations are similar to the properties of other pathogenic mutations, analyses of all cancer mutations together reveal much weaker trends. Despite this, we can observe meaningful effects by considering the collective gene-level properties of cancer mutations. In particular, mutations in tumor suppressors tend to be significantly enriched in structural damage, similar to pathogenic loss-of-function missense mutations. In contrast, mutations in oncogenes tend to be structurally mild but show strong clustering within three-dimensional protein structures, similar to gain-of-function disease mutations. By searching for genes enriched in these mutational properties, we can identify candidate cancer drivers and obtain insight into the molecular mechanisms by which mutations in these genes might act.

The findings here are made possible by the huge number of structural models for human proteins now available. Analyses based on PDB structures are somewhat limited due to the relatively small number of human proteins with published experimentally determined structures. Nevertheless, this PDB-level analysis has allowed us to assess the structural context of 24% of the *cancer-all* missense mutations and 62% of the *cancer-driver* mutations (Table 1). The key advantage of the PDB-based analyses is that they can consider the effects of intermolecular interactions, given the fact that most human proteins are able to assemble into complexes.^78^ Thus, the trends observed for certain proteins were markedly higher when using the PDB structures. For example, TET2 achieves much higher significance in the PDB compared to AlphaFold analysis, likely because the predicted effects of many missense mutations will be greater due to their disruptive effects on the interaction with DNA. In contrast, the AlphaFold models have the advantage of being available for all human proteins, and we note that many of the interesting hits we identified in our search for putative cancer drivers are in proteins for which experimentally determined structures are not available.

A crucial focus of this study has been on predicting the effects of missense mutations on protein stability. However, computationally predicted ΔΔG values are limited in their utility for identifying pathogenic missense mutations compared to evolution-based variant effect predictors (VEPs).^46^ It is possible that using state-of-the-art VEPs rather than ΔΔG values could prove even more powerful for identifying cancer-driving genes. However, we suspect that this strategy may be less informative regarding molecular mechanisms, given that VEPs rely primarily on evolutionary information and should be relatively insensitive to whether or not functionally disruptive mutations are damaging to protein structure.^23^ Given that nearly all VEPs underperform on gain-of-function compared to loss-of-function mutations,^23^ and our observation that cancer-driving mutations appear to be enriched in gain-of-function effects, the ability of the current generation of VEPs to provide insight into cancer-driving mutations and genes may be limited.

Our results provide further demonstration of the utility of our EDC metric, which quantifies the extent of mutation clustering within protein structures, and it has proven valuable for distinguishing between genes associated with loss-of-function, gain-of-function, and dominant-negative disease mechanisms.^23,52,53^ At the same time, it is interesting that no meaningful trends were observed in the *cancer-all* dataset, suggesting that this approach is highly sensitive to the noise associated with passenger mutations. While considering mutation recurrence enabled us to overcome this, it is an imperfect solution that likely loses some useful information. A more nuanced strategy that considers number of occurrences relative to a background mutational null model, as recognized previously by others,^5,79^ may provide a better way of identifying clustering within noisy cancer mutation datasets. Such an approach might also prove useful for identifying more subtle enrichments in structurally damaging mutations, given that not all missense changes are equally probable, as our ΔΔG_rank_ difference approach effectively assumes.

Here, we identified human protein-coding genes that we refer to as candidate cancer drivers. Many of these appear to be interesting, but it is very difficult to confirm any cancer role they may possess. Validating these associations in independent cancer sequencing datasets will be essential for providing further confidence in our predictions. Moreover, by making our gene-level results available, including ΔΔG_rank_ difference and associated *p* values, and recurrent EDC values, we hope that our results will guide others and provide independent evidence in the search for cancer-driving genes.

### Limitations of the study

A key limitation of this study is its focus on pan-cancer effects, which might overlook the nuances of tissue-specific oncogenesis. While this maximizes statistical power for the purposes of this study, we acknowledge that many cancer-driving genes and mutations will have a strong tissue specificity. Tumorigenesis starts as a localized process in a specific tissue, and all the subclonal populations resulting from the original retain features and characteristics of the original cell line. This results in tumors displaying traceable transcriptomes and interactomes, even after metastasis, and distinctive molecular mechanisms linked to their etiology. For instance, approximately 30%–50% of colorectal cancer tumors have a mutated *KRAS* gene,^80^ whereas it has been observed to be mutated in 90% of pancreatic cancers of all grades,^81^ and the mutation signatures differ between them as well. Future work should focus on the tissue specificity of the phenomena observed here, and this will be facilitated by the rapid growth in available cancer sequencing data.

Our analyses using the *cancer-driver* group rely on variant-level CMC classifications, which are based on recurrence, presence in ClinVar, conservation, and signs of positive selection in cancer cells.^35^ In principle, these should be independent of the protein structural information used in our analyses. However, it is possible that structure could influenced some ClinVar classifications, e.g., because a variant was close in space to another pathogenic variant or functional site or because a predicted ΔΔG was high. Thus, there is some potential for circularity in our *cancer-driver* analyses, although we suspect this is very small. Fortunately, however, our results are also supported by the observations in the *cancer-recurrent* group, which should be completely independent of protein structure.

Another potential limitation of this study relates to protein structural bias. Specifically, there is considerable ascertainment bias in the PDB in terms of which proteins have had their structures determined experimentally. The AlphaFold analysis mostly overcomes this issue, given that models are available for all proteins. However, the methodology has been trained on experimentally determined structures, which could lead to some bias toward well-known cancer-associated proteins. Furthermore, while AlphaFold models include intrinsically disordered regions of proteins, they do not represent their dynamic ensemble nature. Recent work on the large-scale modeling of intrinsically disordered ensembles may facilitate a better understanding of their potential roles in cancer.^82^

## Resource Availability

### Lead contact

Queries and further information should be directed to and will be fulfilled by the lead contact, Joseph Marsh (joseph.marsh@ed.ac.uk).

### Materials availability

This study did not generate new unique reagents.

## Star⋆Methods

Detailed methods are provided in the online version of this paper and include the following:

- KEY RESOURCES TABLE
- METHOD DETAILS
  ○ Data collection
  ○ Structural dataset
- QUANTIFICATION AND STATISTICAL ANALYSIS

## Star⋆Methods

### Key Resources Table

**Table:** 

REAGENT or RESOURCE	SOURCE	IDENTIFIER
Deposited data		
Cancer mutation Census	Sondka et al.^35^	N/A
Protein DataBank	Berman et al.^33^	N/A
gnomAD	Karckzewski et al.^37^	N/A
Conservation scores	Zhao et al.^84^	N/A
Experimental protein stability values	Tsuboyama et al.^48^	N/A
Software and algorithms		
R	R Development Core Team^85^	N/A
ChimeraX	Pettersen et al.^86^	N/A
DSSP	Joosten et al.,^87^ Kabsch and Sander.^88^	N/A
FoldX	Delgado et al.^45^	N/A
AlphaFold2	Jumper et al.^34^	N/A

## Method Details

### Data collection

The *cancer-all* dataset was comprised of all somatic missense mutations observed in tumors from the CMC v95,^35^ which was downloaded from the COSMIC portal.^4^ Additional data files were also downloaded for mapping purposes from the same repository, including gene-level classification information and the CGC. The *pathogenic* dataset included missense mutations retrieved from ClinVar as of 2022.10.09, including those classified as pathogenic and as likely pathogenic. The *putatively benign* set included missense variants were collected from gnomAD v2.1.1, excluding any that were present in the pathogenic set. The *cancer-recurrent* dataset only those observed to occur at least seven times in the CMC. Finally, for the *cancer-driver* dataset, we only included the subset of CMC missense mutations directly annotated as having a role in cancer. Although these mutations are assigned a tier level of 1–3 in the CMC based on strength of evidence, we grouped them all together here due to the limited size of our dataset.

Only gene level classifications of “oncogene” and “TSG” were considered. Although the CMC categorises some genes as being associated with “fusion”, we ignored this given that it represents a fundamentally different type of genomic change compared to the missense mutations we are interested in. Thus, a gene classified in the CGC as “fusion” was considered as having no role in our analysis, while a gene classified as “oncogene, fusion” would be considered an oncogene.

### Structural dataset

The PDB analysis was performed using the same analysis pipeline as previously described.^23^ In short, protein structures were downloaded from the Protein DataBank on 2022.08.05, using the first biological assembly for each entry. All missense mutations were mapped to structures, considering regions with >90% sequence identity to the human protein over a region of at least 50 residues. In cases where a residue maps to more than one polypeptide chain, we first selected the highest resolution structures, and in the case of ties, selected the largest biological assembly. The AlphaFold analysis used AlphaFold2 version 1 models,^34^ downloaded from https://alphafold.ebi.ac.uk/download on 2021.07.27. Secondary structure of each residue was classified with DSSP,^88^ and interior, surface and interface residues were defined according to relative solvent accessibility (RSA).^83^ “Interior” residues have an RSA ≤0.3; “Interface” residues have an RSA between 0.3 and 0.5 (0.3 < RSA <0.5); “Surface” residues have an RSA ≥ 0.5.

## Quantification And Statistical Analysis

FoldX 5.0 calculations were performed using all default parameters, with three replicates per mutation, and the ‘RepairPDB’ function run in advance. Only ‘full’ ΔΔG values based on the entire biological assembly were used for PDB structures, while AlphaFold models were monomeric. For large proteins, where multiple overlapping AlphaFold models are generated, we averaged ΔΔG values over all available models for each variant. We rank normalise absolute ΔΔG values to obtain the ΔΔG_rank_ metric, whereby the mildest |ΔΔG| is defined as being equal to 0, the highest |ΔΔG| was defined as 1, and the mean of all possible amino acid substitutions in a protein was equal to 0.5. Absolute ΔΔG values were used, based on our previous observation of their slightly improved correspondence with mutation pathogenicity.^46^ Gene-level evolutionary conservation values for comparison were obtained by averaging residue-level conservation scores across all residues from the DescribePROT database.^84^

To calculate the Extent of Disease Clustering (EDC) metric,^23^ for each residue in a protein subunit, we obtain the Cα:Cα distance *D* to all other residues in the same subunit with disease mutation in the relevant dataset, and the closest distance *D*_*min*_ is selected. We calculate the average of the log distance $\left(\bar{D}\right)$ for all disease residues, and all non-disease residues separately as

$$\bar{D}=\frac{1}{n}\overset{n}{\underset{i=1}{\sum }}\log\left(D_{\min}\right)$$

The EDC is the ratio of the two values:

$$EDC=\frac{{\bar{D}}_{non-disease\;}}{{\bar{D}}_{disease\;}}$$

For PDB structures, all residues were considered. However, for AlphaFold models, residues with low-confidence structural predictions, having predicted local distance difference test (pLDDT) values less than 70, were excluded from the calculation. This is similar to our most recent studies,^52,53^ as we found that, for pathogenic missense mutations, this results in much better discrimination between proteins with loss-of-function and non-loss-of-function mutations when using EDC derived from AlphaFold models. Only proteins with mutations occurring at five or more residues were considered. For the recurrent analysis, only residues observed to have been mutated at least seven times (using the COSMIC_SAMPLE_MUTATED column in the CMC dataset) were included. Our results are robust to the choice of these thresholds (Figure S5).

All data curation, mapping and statistical analysis was carried out using R. RStudio was used for scripting. All three datasets mentioned in the data collection section were filtered keeping only unique missense mutations to avoid potential biases and duplicates in the results. The collection of R packages from the tidyverse and the data.table package were used to smooth and speed up the running time of the code, as well as to significantly increase the legibility of the code. The furrr and future packages were used to implement parallel computing and optimise code runtime. Data visualisation was achieved using the R package ggplot2 and extensions based on it, namely ggstatsplot (for in-plot statistics), ggrepel (for non-overlapping labeling), and patchwork (for composing multi-pane plots). ChimeraX^86^ (v1.5) was additionally used to visualise variant clustering in a 3D context. Some discrepancies and inconsistent annotations resulted in dropping a very small number of mutations for each dataset (≈1% variant loss for the three databases), mostly found in fusion genes. The upper and lower whiskers of all boxplots are defined by the ggplot2 plotting function.

Pairwise comparisons were carried out using two-sample Wilcoxon tests (also known as Mann-Whitney tests), and the statistical significance defined as *p* ≤ α using a traditional significance level of α = 0:05. The statistical significance threshold was modified using a Bonferroni correction on the per-protein comparisons individually for PDB structures and the AlphaFold models as *p* ≤ α/*N*, where *N* is the number of observations (proteins in this case): *N* = 8402 for the analysis on PDB structures and *N* = 17905 for the analysis on AlphaFold models. The analysis of categorical variables was carried out using the Fisher exact test, setting a traditional statistical significance of *p* ≤ 0.05.

## Supplementary Material

Supplemental information can be found online at https://doi.org/10.1016/j.celrep.2024.114905.

Document S1

## Figures and Tables

**Table 1:** Number of protein-coding genes and missense mutations present in the different datasets used in this study, considering those present in PDB structure or AlphaFold models

		PDB structures			AlphaFold models	
Dataset		Genes	Mutations		Genes	Mutations
*Putatively benign*		9,029	1,161,381		19,171	5,607,699
*Cancer-all*		8,402	643,008		17,905	2,675,629
*Cancer-recurrent*		1,781	5,007		6,576	17,665
*Cancer-driver*		624	2,080		1,087	3,348
*Pathogenic*		2,198	30,271		3,940	47,697

## Data Availability

Complete datasets associated with the analyses in this study are available at https://osf.io/vk68d/. The original code for this paper can be found in the same repository as the datasets. Any additional information required to reanalyze the data reported in this work paper is available from the lead contact upon request.

## Highlights

- Analyzed protein structural context of mutations to reveal cancer-driving mechanisms
- Identified distinct structural patterns in tumor suppressors and oncogenes
- Predicted candidate cancer driver genes using structural and clustering metrics

## In brief

Chillón-Pino et al. investigate the structural context of cancer mutations across thousands of human proteins, revealing distinct patterns in tumor suppressors and oncogenes. Their study identifies cancer driver genes by analyzing predicted impacts on protein stability and clustering patterns of mutations.
