# Supplementary material for: Protein structural context of cancer mutations reveals molecular mechanisms and candidate driver genes
Source: Cell Rep. Author manuscript; Available in PMC 2025 Mar 27. (PMC7617530; doi:10.1016/j.celrep.2024.114905)
Supplement: Document S1 [file EMS204024-supplement-Document_S1.pdf]

Cell Reports, Volume *43*

Supplemental information

Protein structural context of cancer mutations reveals molecular mechanisms and candidate driver genes

Diego Chillón-Pino, Mihaly Badonyi, Colin A. Semple, and Joseph A. Marsh

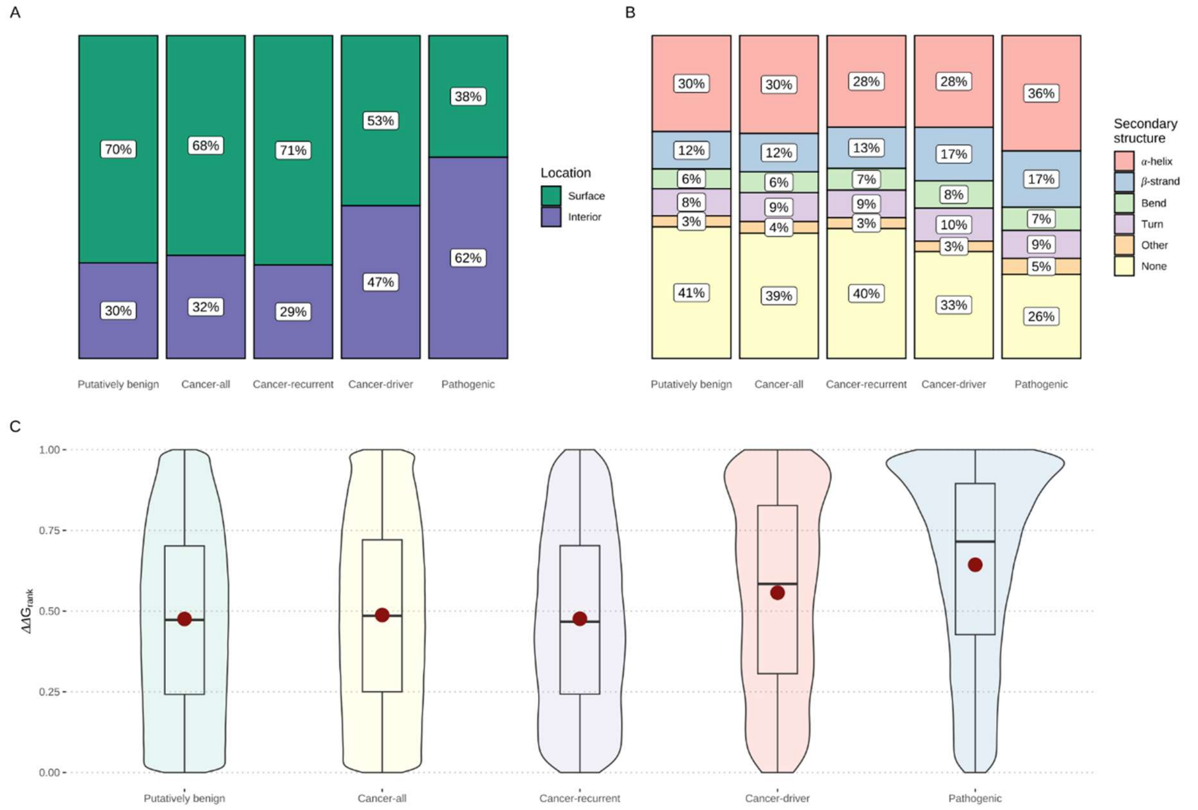

Figure S1 | Protein structural properties of different types of missense mutations based on AlphaFold models, related to Figure 1. Putatively benign mutations are those observed in the human population (gnomAD) without a reported disease association. *Cancer-all* are all cancer-associated mutations from the CMC. *Cancer-recurrent* comprises only recurrent mutations from the CMC (recurrence ≥ 7). *Cancer-driver* mutations are the subset of cancer mutations annotated for their direct role in cancer. Pathogenic mutations are those annotated as pathogenic or likely pathogenic in ClinVar. **(A)** Locations of mutations within protein structures in AlphaFold models. **(B)** Occurrence of mutations within different types of secondary structures. **(C)** Violin plot distributions of predicted structurally damaging effects, as measured by the $\Delta\Delta G_{rank}$ metric, whereby 0 represents the mildest possible single amino acid substitution in a protein, 1 represents the most damaging, and random mutations would be expected to have a mean of 0.5. The mean value of each distribution is represented with a red dot for the datasets. All iterations of group pairs are highly significantly different (p-value $< 7.9 \times 10^{-8}$) according to Wilcoxon tests, except between the *putatively benign* and *cancer-recurrent* groups.

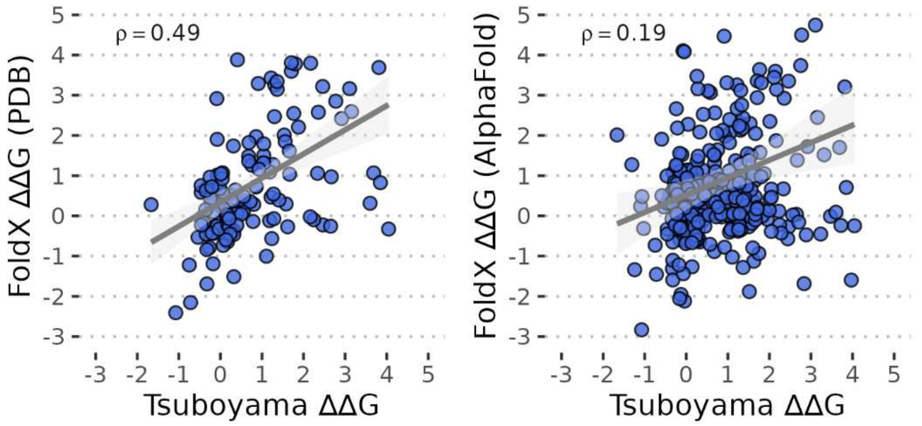

Figure S2 | Correlation or computationally predicted ΔΔG values with high-throughput experimental values, related to Figure 1. Experimental values form Tsuboyama *et al.* showed a moderate Spearman correlation (ρ) with FoldX calculated ΔΔG values based on experimental structures (left). In contrast, the correlation is markedly lower for ΔΔG values calculated from AlphaFold models, which is likely due to the presence of disordered regions.

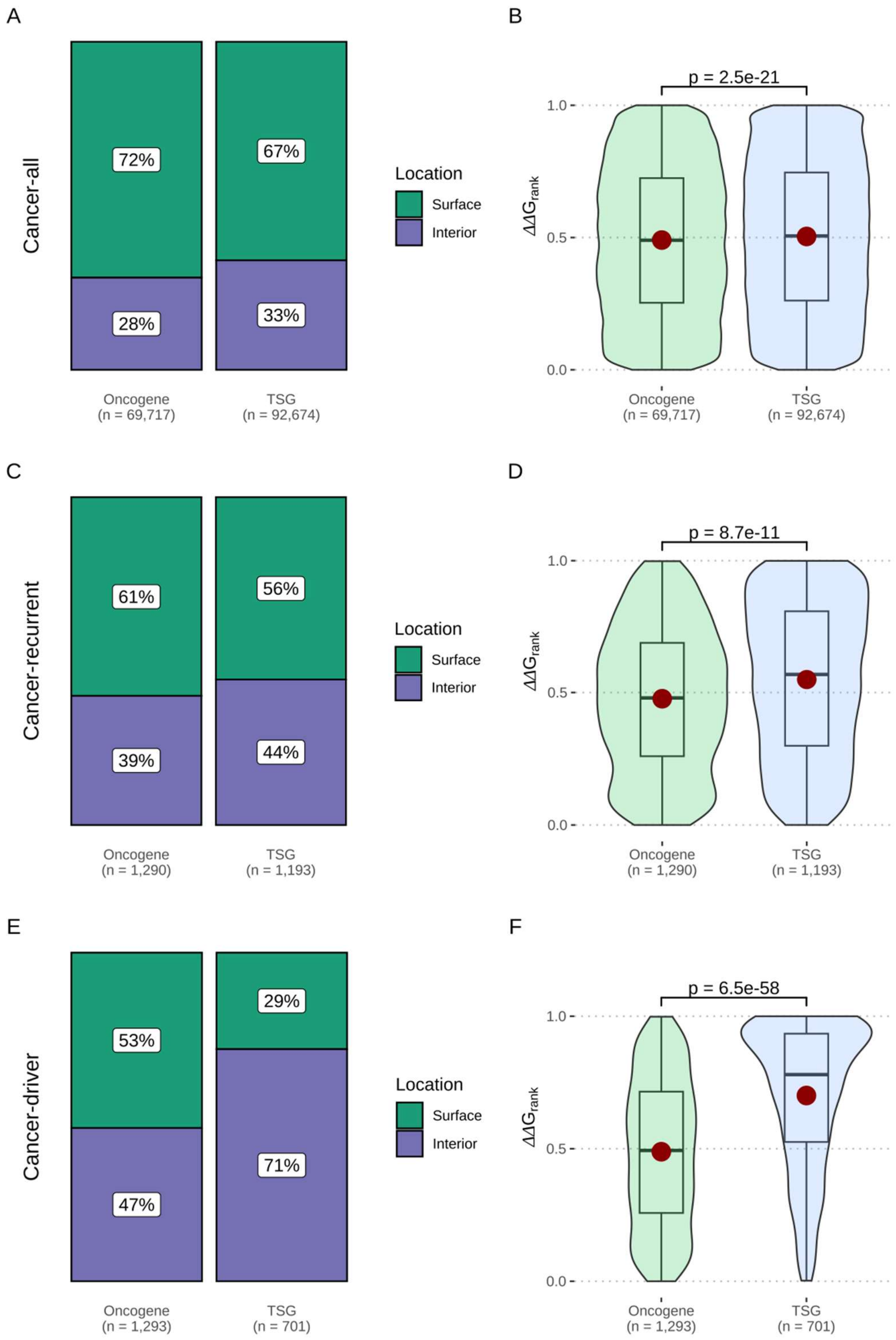

Figure S3 | Protein structural properties of cancer mutations in oncogenes and tumour suppressors, based on AlphaFold models, related to Figure 2. (A) Locations of all cancer-associated mutations (*cancer-all* dataset) and **(B)** distributions of predicted structural damage, as represented by $\Delta\Delta G_{rank}$ values. **(C)** Locations of *cancer-recurrent* mutations models (cancer-recurrent dataset) and **(D)** distributions of predicted structural damage, as represented by $\Delta\Delta G_{rank}$ values. **(E)** Locations of *cancer-driver* mutations and **(F)** distributions of $\Delta\Delta G_{rank}$ values. The *p*-values are calculated using Wilcoxon tests.

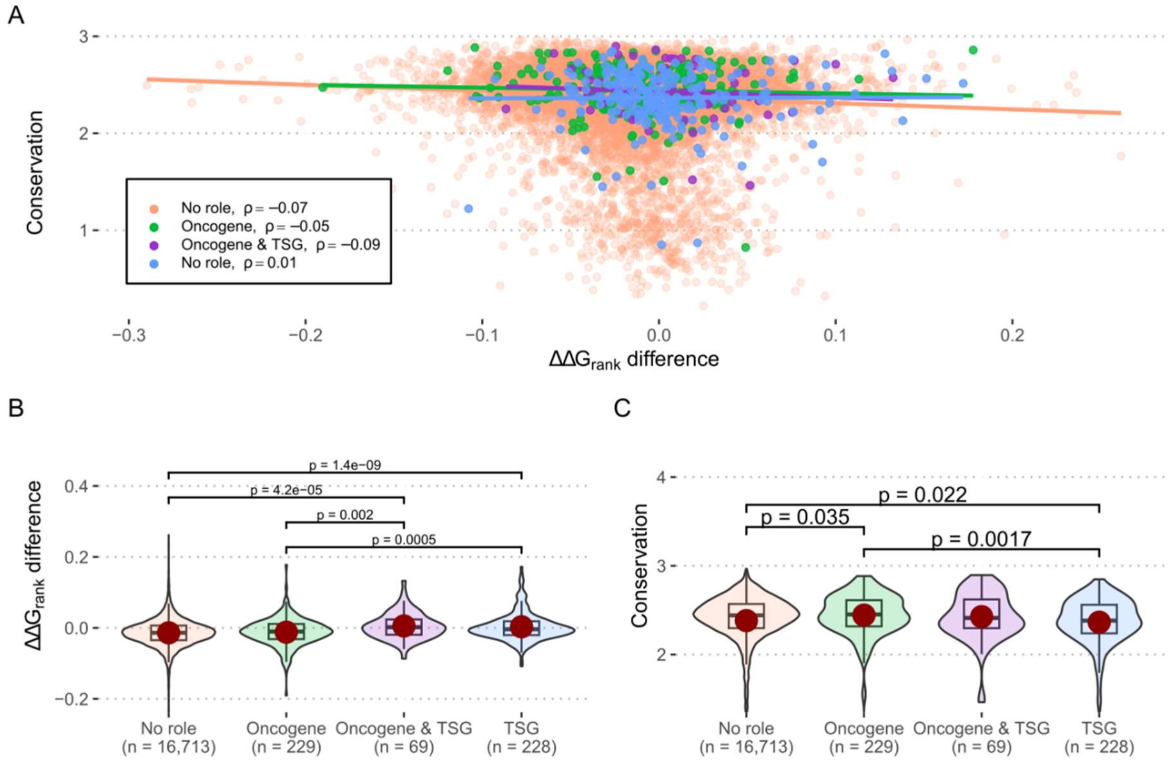

Figure S4 | Relationship between evolutionary conservation and structural damage in human proteins, related to Figure 3. (A) Correlation between $\Delta\Delta G_{rank}$ difference values calculated from AlphaFold models and mean conservation score per protein, with Spearman correlation (ρ) values. **(B)** Distribution of $\Delta\Delta G_{rank}$ difference values across different gene glasses. **(C)** Distribution of evolutionary conservation values across different gene classes. P-values are from Wilcoxon rank-sum tests, and *p*-values < 0.05 values are shown. Accounting for multiple testing, the no role *vs* oncogene and no role *vs* TSG comparisons do not remain significant.

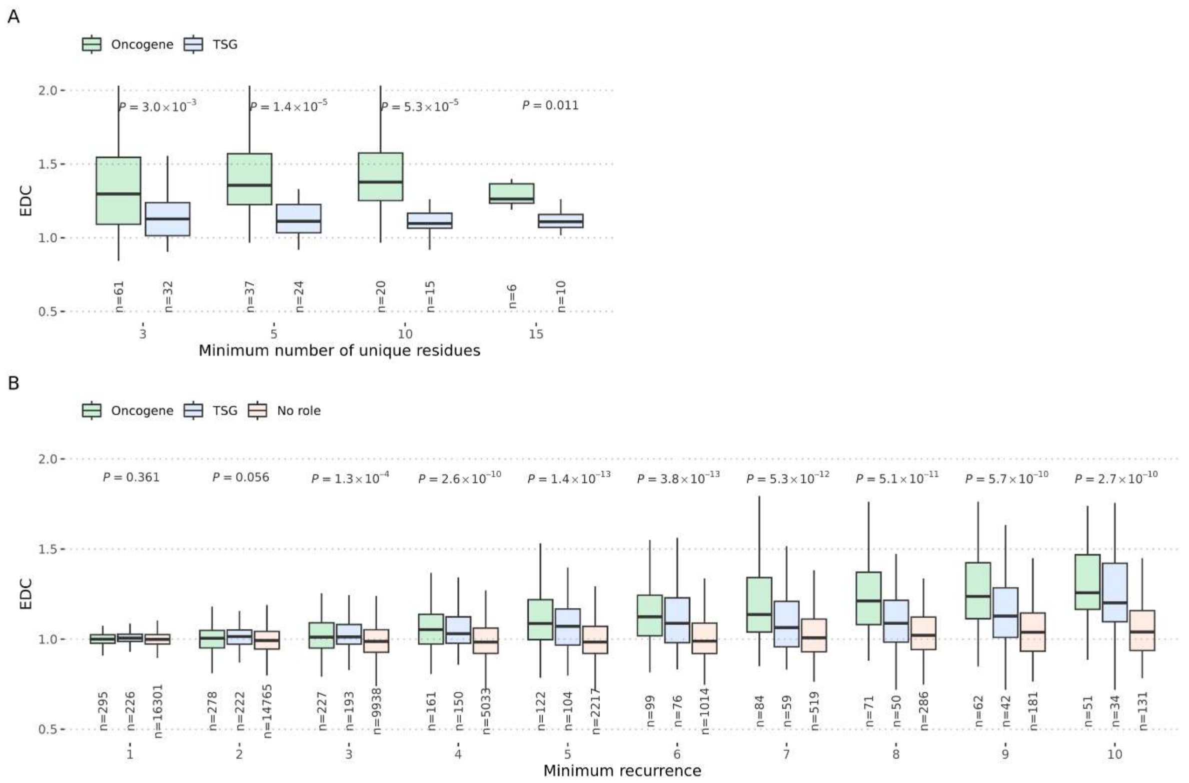

Figure S5 | Thresholds for the number of unique and recurrent residues for calculating EDC, related to Figure 4. (**A**) The effect of the minimum number of unique residues on EDC values (at a minimum recurrence of 7).*P*-values were calculated with the Wilcoxon rank-sum test. (**B**) The effect of minimum recurrence (number of times a residue has been observed to be mutated in cancer samples) on EDC values (at a minimum number of unique residues >=5). *P*-values are given for comparisons between "Oncogene" and "No role" and were calculated with the Wilcoxon rank-sum test. Sample sizes at the bottom represent the number of genes in each group.

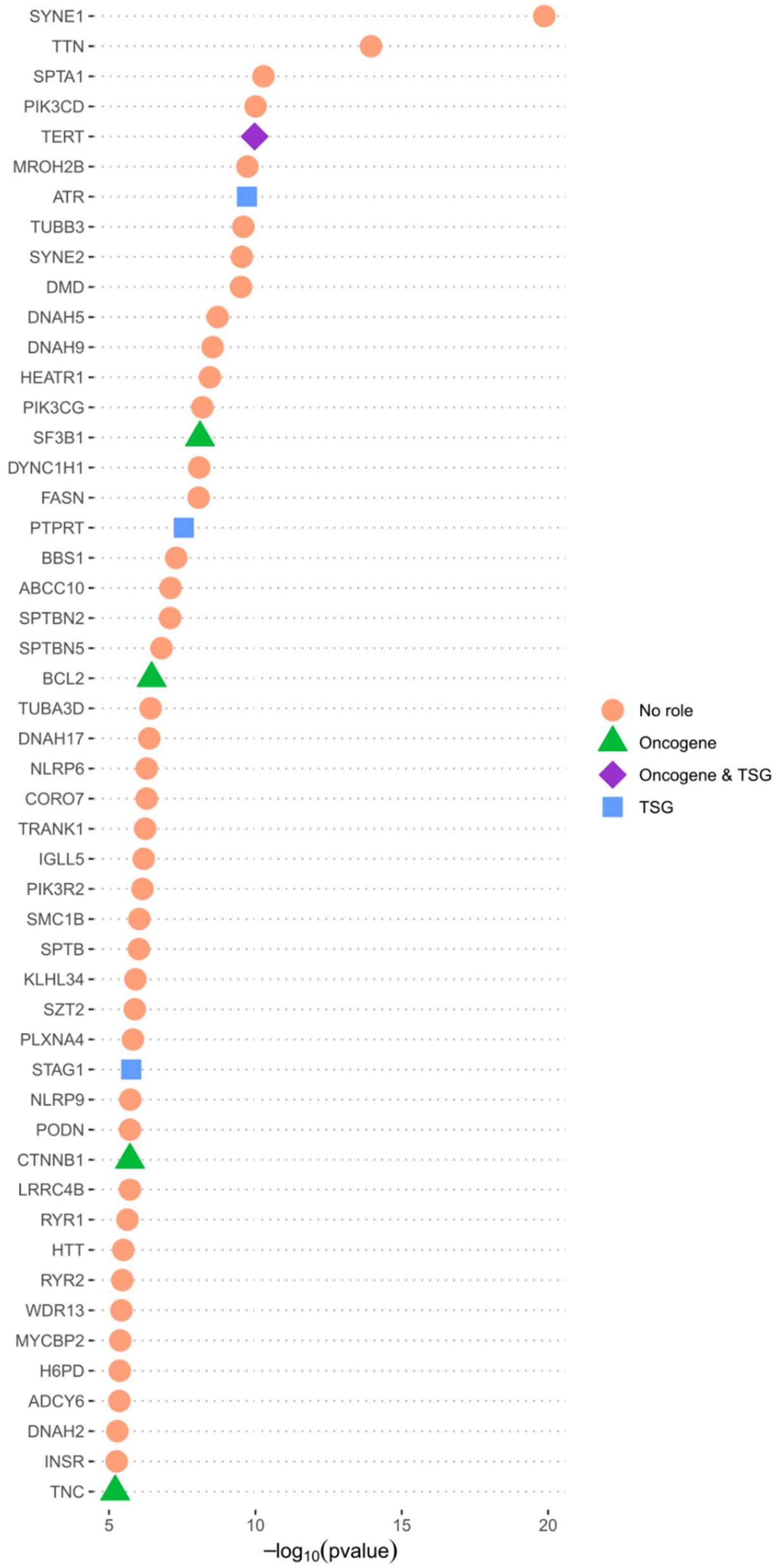

Figure S6 | Top 50 human protein-coding genes enriched in structurally mild mutations, related to Figure 5. These are proteins with negative $\Delta\Delta G_{rank}$ difference values (*i.e.* those on the left side of the volcano plots in **Fig. 3**), for which the average observed missense mutation in the *cancer-all* dataset is less destabilising than the average unobserved but possible mutation. *P*-values from both the PDB and AlphaFold analyses in **Fig. 3** are included, with the most significant value from either analysis selected for each protein to be used in this ranking.
